# A randomized controlled trial testing the effectiveness of a paramedic-delivered care transitions intervention to reduce emergency department revisits

**DOI:** 10.1186/s12877-018-0792-5

**Published:** 2018-05-03

**Authors:** Ranran Mi, Matthew M. Hollander, Courtney M. C. Jones, Eva H. DuGoff, Thomas V. Caprio, Jeremy T. Cushman, Amy J. H. Kind, Michael Lohmeier, Manish N. Shah

**Affiliations:** 1 Department of Emergency Medicine, University of Wisconsin School of Medicine and Public Health, Madison, Wisconsin USA; 2 Department of Emergency Medicine, University of Rochester Medical Center, Rochester, NY USA; 3 Department of Health Services Administration, University of Maryland-College Park, Baltimore, Maryland USA; 4 Department of Medicine (Geriatrics), University of Rochester Medical Center, Rochester, NY USA; 5 Department of Medicine (Geriatrics and Gerontology), University of Wisconsin School of Medicine and Public Health, Madison, Wisconsin USA; 6 VA Geriatrics Research Education and Clinical Center, Madison, USA

**Keywords:** Care transitions, Community paramedicine, Emergency department, Older adults

## Abstract

**Background:**

Approximately 20% of community-dwelling older adults discharged from the emergency department (ED) return to an ED within 30 days, an occurrence partially resulting from poor care transitions. Prior published interventions to improve the ED-to-home transition have either lacked feasibility or effectiveness. The Care Transitions Intervention (CTI) has been validated to decrease rehospitalization among patients transitioning from the hospital to the home but has never been tested for patients transitioning from the ED to the home. Paramedics, traditionally involved only in emergency care, are well-positioned to deliver the CTI, but have never been previously evaluated in this role.

**Methods:**

This single-blinded randomized controlled trial tests whether the paramedic-delivered ED-to-home CTI reduces community-dwelling older adults’ ED revisits in the 30 days after an index visit. We are prospectively recruiting patients aged≥ 60 years at 3 EDs in Rochester, NY and Madison, WI to enroll 2400 patient subjects. Subjects are randomized into control and treatment groups, with the latter receiving the adapted CTI. The intervention consists of the paramedic performing one home visit and up to three follow-up phone calls. During these interactions, the paramedic follows the CTI approach by coaching patients toward their goals, with a focus on their personal health record, medication management, red flags, and primary care follow-up. We follow patient participants for 30 days. All receive a survey during the index ED visit to capture baseline demographic and health information and two telephone-based surveys to assess process objectives and outcomes. We also perform a medical record review. The primary outcome is the odds of ED revisit within 30 days after discharge from the index ED visit.

**Discussion:**

This is the first study to test whether the CTI, applied to the ED-to-home transition and delivered by community paramedics, can decrease the rate at which older adults revisit an ED. Outcomes from this research will help address a major emergency care challenge by supporting older adults in the transition from the ED to home, thereby improving health outcomes for this population and reducing potentially avoidable ED visits.

**Trial registration:**

ClinicalTrials.gov Registration: NCT02520661. Trial registration date: August 13, 2015.

## Background

The emergency department (ED) is an important provider of acute medical care to the 43 million older adults (age ≥ 65) residing in the United States [1]. In 2013 alone, older adults made 20.8 million ED visits [2]. However, such figures are misleading, as approximately 20% of ED use among older adults is actually a *revisit,* in which a discharged patient returns within 30 days of the index visit either for the same complaint or a new unrelated issue [3–8]. Compared to younger populations, older adults experience a higher frequency of revisits and adverse health outcomes following discharge [9].

Process measures potentially explaining these outcomes point to poor care transitions [10]. Older adults too often leave the ED without adequate understanding of discharge instructions such as how to manage medication changes, follow up with their primary care physician (PCP), and recognize red flags or illness warning signs necessitating immediate attention. Although ED personnel typically provide verbal and written instructions, 78% of patients across all age groups display deficient comprehension, and the discharge process lasts, on average, only 4 min [11, 12].

Efforts to reduce avoidable ED use by older adults have therefore focused on improving their relatively difficult transition from ED to home [13–20]. Unfortunately, prior published attempts have encountered problems of program feasibility or effectiveness. For example, the discharge planning and follow-up program of Guttman and colleagues, which required an average of 30 min per patient during the ED stay, improved patient satisfaction but did not show a statistically and clinically significant reduction of ED revisits within 14 days [14].

We seek to improve the ED-to-home transition for community-dwelling older adults by applying a slightly modified Care Transitions Intervention (CTI) to those individuals being discharged home from the ED. The original, hospital-to-home CTI is a validated and widely implemented program. Using the hospital-to-home CTI decreased the 30-day rehospitalization rate from 11.9 to 8.3% (*p* = 0.048) and the 90-day rehospitalization rate from 22.5 to 16.7% (*p* = 0.04) in the original validation studies. Furthermore, Coleman and colleagues reported that the mean hospital costs were reduced for CTI patients ($2058 vs. $2546, *p* = 0.049) at 180 days [21–23]. However, the model has not been tested for the ED-to-home transition.

Specifically, the CTI model consists of a structured, 4-week program in which a trained coach, originally an advanced practice nurse, provides one in-person visit in the hospital, one in-person home visit, and up to three phone calls. The coach uses motivational interviewing techniques, behavior modelling, skill transfer, and role playing to enhance patients’ abilities to ensure effective medication management, PCP follow-up, red flag awareness, and maintenance of a personal health record. Coaches do not directly provide services (e.g., make appointments, deliver health care).

By adapting the CTI to the ED-to-home transition, we hope to translate the benefits of this model of care to the ED setting. Furthermore, instead of using advanced practice nurses, we deliver the CTI program through paramedics from the ambulance-based emergency medical services (EMS) system [24–26]. These individuals comprise an underused, highly skilled, and highly respected resource present in all communities, and have been increasingly integrated into providing community health interventions, a mission beyond their traditional focus on emergency care.

The primary goal of this study is to test the effectiveness of the paramedic-delivered, modified CTI Program using a randomized controlled trial design. We are evaluating the overall hypothesis that CTI Program participants will have lower odds of ED revisits within 30 days of discharge from the ED compared to control participants. Secondary hypotheses include the propositions that compared to control participants, those receiving the intervention will: 1) have increased patient activation 30 days after discharge from the ED; 2) have shorter time to follow-up with their physician; 3) implement medication changes within 4 days of ED discharge; and 4) have lower healthcare costs within 30 days of discharge from the ED. This protocol description outlines how the CTI Program was modified for this specific patient population and how the study will test these hypotheses.

## Methods

This study is a single-blinded randomized controlled trial. The Institutional Review Boards (IRB) at the University of Wisconsin-Madison and the University of Rochester approved this study. The trial was registered at ClinicalTrials.gov (NCT02520661, registration date: August 13, 2015) [27, 28]. Any significant protocol modifications will be reported to these IRBs.

Our three specific aims are as follows:

1. Assess the process outcomes of our ED-to-home CTI program for older adults treated in the ED and discharged home, as compared to usual care. The outcomes measured consist of the following: patient’s understanding of ED discharge instructions, implementation of medication changes, and time to follow-up with a primary care physician.
2. Determine the effectiveness (e.g., patient activation, ED revisit rate) and cost-effectiveness (e.g., healthcare costs) of our ED-to-home CTI program for older adults treated in the ED and discharged home, as compared to usual care.
3. Identify biomedical (e.g., age, comorbidities, impaired cognition, functional limitation) and psychosocial (e.g., social connectedness, anxiety, depression) factors independently associated with repeat ED visits within 30 days of ED discharge among ED-to-home CTI program participants.

### Designing the program

Modifying the CTI to apply it to the ED-to-home transition necessitated the design and refinement of two structural elements: community paramedic coach training, and the CTI Program.

#### A pragmatic application of the care transitions intervention program

For our ED-to-home CTI, we sought to make the fewest possible changes to the CTI. Following discussions among the research team members, comprising CTI experts, geriatricians, emergency physicians, EMS physicians, and paramedics, we decided to make two overall changes. These modifications are primarily driven by pragmatic considerations. By retaining other CTI features we pursue a balance between changes necessary for success in the ED setting and preservation of characteristics that have made the CTI successful.

We first determined that the in-person coach visit in the ED would be impractical due to time constraints in that setting. A prolonged discharge process would limit widespread implementation and sustainability since EDs are very busy and need the space for new patients. Therefore, we chose to briefly introduce the program to patients during the ED visit and ensure that the home visit rapidly follows ED discharge, ideally within 24–48 h [29]. During this visit and subsequent follow-up phone calls, the coach would work with the patient to achieve his or her goals through the Four Pillars of the program (Table 1).

**Table 1:** Model of the modified care transitions intervention: the Four Pillars

Pillar	Medication self-management	Follow-up	Red flags	Patient-centered health record
Goal	Know medications & have system to take them	Schedule appropriate follow-up visits	Know indications that a condition is worsening and how to respond	Understand and manage a personal health record
Home Visit	Discuss importance of knowing medications Reconcile medications; correct discrepancies	Emphasize importance of follow-up visit; practice and role-pay questions for the PCP	Discuss symptoms and side effects of medications	Explain the personal health record Review discharge summary; encourage patient to share health record with PCP
Follow-up calls	Answer any remaining medication questions	Advocate for getting an appointment, if needed	Reinforce when/if PCP should be called	Discuss outcome of PCP visit

**Fig. 1:**
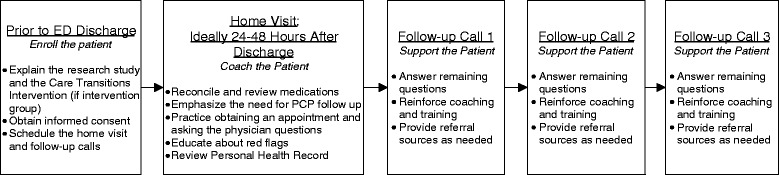
Emergency Department-to-Home Care Transitions Intervention

Second, we chose to deliver the program through paramedics instead of advance practice nurses, as used in the original studies by Coleman and colleagues. We see paramedics as a viable alternative as the ED-to-home CTI program requires robust infrastructure, with home visits needing to be made in urban, suburban, and rural communities, on any day of the week. The broader availability of the ambulance-based emergency medical services (EMS) system fits such demanding requirements, making paramedics optimal agents for program delivery. Figures 1 summarizes the ED-to-home CTI Program.

**Fig. 2:**
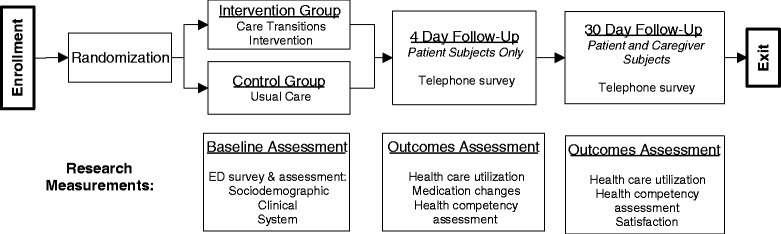
Overview of Research Activities: Participant Flow, Actions, and Measurements

#### Developing community paramedic coach training

To deliver the CTI, community paramedics must effectively coach and motivate patients. Thus, we provide participating paramedics the necessary training to successfully function as CTI Program coaches. The training consists of readings, video podcasts, and experiences related to the CTI Program, including the training from the Care Transitions Program at the University of Colorado (Denver, CO). Paramedics also receive training in motivational interviewing, geriatrics, the ED discharge process, and community paramedicine. Experiences include CTI coach, emergency physician, and geriatrician shadowing; simulation; and mentored coaching. Because we use paramedics, we do not feel the need to provide medical education other than in aging-specific topics. We have published an evaluation of this training program [30].

### Setting

We are performing this study (Fig. 2) at the EDs of three hospitals (two in Rochester, New York and one in Madison, Wisconsin). The three sites are University Hospital at the University of Wisconsin-Madison; Strong Memorial Hospital and Highland Hospital at the University of Rochester.

### Eligibility

Both patients and their caregivers are eligible to participate in the study. Patient subjects must be age 60 years or older, speak English, reside in Dane County, Wisconsin or Monroe County, New York, and have a primary care physician affiliated with UWHealth or UR Medicine. We limited patients to these two health systems to maximize the quality of data collection from the electronic health record (EHR). Furthermore, patient subjects must live in independent home dwellings, operationalized as not living in skilled nursing facilities or assisted living facilities, have a working telephone, and have the ability to provide informed consent (or have a legally authorized representative provide consent). Finally, patient subjects must be discharged from the ED, including ED observation, within 24 h of arrival, as both of those transition processes are similar. We exclude patients if they have participated in the study previously; are actively enrolled in hospice, a transitions program, or an intensive care management program; are homeless; present for a behavioral health problem; or are severely ill, as defined by an Emergency Severity Index category of 1 [31]. Caregiver subjects must be adults, speak English, have a telephone, and provide informed consent.

### Recruitment and enrollment

By monitoring the Epic (Verona, WI) ED track board, research staff identify potentially eligible patients. For each potentially eligible patient, the staff member then queries the patient’s healthcare provider to confirm that the patient will likely be discharged home. If the healthcare provider indicates that this outcome is likely, a staff member then approaches the patient and family/informal caregivers, if any, to confirm eligibility and obtain consent. Caregivers are only consented after the patient subject is consented. At times, this process results in consenting patients later becoming ineligible because they are not discharged home.

Since the study population consists of older adults who may have diminished decisional capacity, we follow a conservative approach and assess each subject’s (patient and caregiver) capacity to provide informed consent. In cases of patients who display a limited capacity to consent, a surrogate (legally appointed representative) can provide consent. We exclude caregivers lacking capacity.

When eligible patients in the ED consent to participate, they receive randomization into either the adapted CTI (intervention group) or usual care (control group). To randomize each participant in a blinded fashion, we provide research staff members with sealed and opaque envelopes, numbered sequentially. Staff members open the envelopes at the completion of the baseline survey at the end of enrollment. Randomization is performed in blocks of 20. The research staff members who enroll the subjects also notify the paramedics as to the scheduled home visit. To maintain blinding, the staff member who enrolls the subject is different than the staff member who performs the telephone follow up. All records regarding the intervention are segregated from the primary study data forms to minimize accidental unblinding of subjects’ study status.

For both groups, we perform research assessments at baseline (in the ED) and again by phone 4 and 30 days after discharge. Figure 2 outlines the research activities.

We took a number of steps to enhance recruitment and participation. First, we provide intervention group patients an appointment card with their community paramedic coach’s picture to address security concerns. Second, we preschedule the follow-up phone calls for patient and caregiver subjects. Third, we collect alternate contact numbers from patients and caregivers, so we can call the alternate number if the subject does not answer the primary phone number. Finally, we have a structured system to obtain follow up, including calls at different times of the day during a short follow up window.

### Data collection

All participants regardless of intervention assignment are assessed in the ED to obtain baseline characteristics, and again assessed 4 and 30 days, respectively, after ED discharge via phone calls delivered by research assistants. Caregivers, if enrolled along with the patient, are assessed only in the ED and at 30 days. For the intervention group, coaches complete surveys after the CTI home visits and phone calls to quantify the coaching interactions and the coaches’ perceived value of the interactions.

Finally, we review the patient’s EHR for a 60-day period—30 days prior to and 30 days after the enrollment in the ED. Information extracted include comorbidities, medications, ED care, and ED discharge instructions. Table 2 outlines the study’s measures, the times at which the information is collected, and the source of the data.

**Table 2:** Study measures: demographic, clinical, covariate, and outcomes

	Measures		
	Measures	Timeline	Source/Approach
Demographic	Patient age, gender, marital status, race, ethnicity, education level, primary language, home ownership, living status, home address	Baseline	Patient survey
	Patient relationship with PCP	Baseline	Patient survey
	Insurance plan	Baseline	Chart review
Clinical	New home services (since ED visit)	Day 4/Day 30	Patient survey
	ED chief complaint, final diagnosis (ICD-10), discharge medications, and instructions	Baseline	Chart review
	CTI Coaching & Services Forms Personal Health Record (PHR) Home Visit/PHR Discussion Checklist Follow-up Phone Calls Checklist Patient Activation Assessment Medication Discrepancy Tool Care Transitions Measure - 3 [40]	Home visit & coach follow up calls	CTI program records
Covariates	Medical history, including Charlson Comorbidity Index [41]	Baseline	Patient survey
	Healthcare: Medications, home services, ED / hospital use	Baseline	Patient survey/ Epic review
	Health Status: Short Form-12 [42]	Baseline	Patient survey
	Disability Status: ADL [43]	Baseline	Patient survey
	Cognition: Short Blessed Test [44, 45]	Baseline	Patient survey
	Social Isolation: PROMIS Social isolation short form [46]	Baseline	Patient survey
	Depression: PHQ-9 [47]	Baseline	Patient survey
	Anxiety: GAD-2 [48]	Baseline	Patient survey
	Health literacy	Baseline	Patient survey
Outcomes	Family Caregiver Activation in Transitions [49]	Baseline, Day 30	Caregiver survey
	Understanding of red flags	Day 4	Patient survey
	Medication changes implemented	Day 4	Patient survey
	Follow up with PCP, specialists, urgent care	Day 4, 30	Patient survey / Chart review
	Wallston’s Perceived Health Competence Scale (PHCS) [50]	Baseline, Day 30	Patient / Caregiver survey
	Healthcare use within 30 days of discharge	Day 30	Patient survey / Chart review
	Death within 30 days of discharge (Social Security Death Index)	Day 30	Death Index
	Patient experiences of continuity	Day 30	Patient survey
	Cost of healthcare and CTI program	Day 30	Health systems/ CTI program records
	Program satisfaction	Day 30	Patient / caregiver survey

To maximize data quality, we split the tasks of data collection and quality assurance among different individuals. A research staff member initially records all data for a given patient and caregiver on paper forms, whether the original source comes from a survey or chart review. Later, a different staff member reviews the forms for quality assurance. When possible, a third staff member enters the data into REDCap, a secure web application for building and managing online surveys and databases.

For research quality assurance purposes, we track a number of metrics. First, the screening of, enrollment of, and application of study instruments to subjects through the study, as presented in Fig. 2, is tracked on a weekly basis. By evaluating the proportion of subjects eligible who are approached to participate in the study, the proportion of subjects consenting to participate, and the proportion of subjects who become ineligible after consent, study processes can be modified to maximize enrollment. Furthermore, querying the reasons for refusing to participate allows identification of consistent themes that can then be addressed during enrollment. We also monitor coaching activity through a services inventory log. By tracking the proportion of subjects who receive the various aspects of the CTI, we can ensure complete delivery of the CTI Program. Finally, we have a rigorous data quality assurance process, using automated and manual checks, to ensure data collected and entered into REDCap are accurate.

### Sample size

This study is designed to have adequate power to test the primary hypotheses that the CTI will result in lower odds of repeat ED use within 30 days of discharge compared to the control group. Based on published reports and local data obtained during the grant submission, we expect 20% of the subjects in the control group to have at least 1 repeat ED visit within the 30-day follow-up period. To detect a 5% absolute decrease in the frequency of repeat ED visits with 80% power using a Chi-square test at a two-sided significance level of 5%, we will need 860 subjects per group. We anticipate approximately 25% attrition over the 30-day follow up period. As such, a final sample size of 1200 subjects in each group will be recruited for participation, making a total target of *N* = 2400. We also calculated the minimum detectable effect size for testing individual biomedical and psychosocial factors predicting repeat ED visit in Aim 4 for the intervention group only. With an expected 15% baseline proportion of repeat ED visit, the sample size of 860 in the intervention group will have 80% power to detect an odds ratio (OR) of 1.3 per standard deviation of a normally distributed predictor. Similarly, 80% power is available to detect an OR ranging from 1.6–1.7 for a binary predictor prevalent in 30–50% of subjects.

### Data analysis

We will use multivariate regression models to examine the effect of program participation on outcomes while controlling for patient-level confounding factors. To adjust for potential baseline differences between intervention and control subjects, we will construct a multiple logistic regression model, with repeat ED use (then other outcomes) as the dependent variable, intervention group as the primary independent variable, and any covariates that were found to be imbalanced at baseline in our analyses. We will also account for clustering of study subjects by state and ED enrollment site.

The proposed economic assessment considers the financial costs and benefits of the CTI. We include fixed and variable costs in our analysis while excluding costs associated with the research. We will make economic projections of the program’s financial sustainability and scalability. We will conduct a series of sensitivity analyses by modifying local wages, population case-mix and program effectiveness to estimate boundaries to program’s sustainability and optimal operational conditions.

We will also identify predictors of repeat ED use among intervention group subjects. To evaluate the relationship between biomedical and psychosocial factors with the occurrence of repeat ED visits among intervention subjects, we will conduct bivariate analyses between patient characteristics and the primary outcome measure (repeat ED visits within 30 days).

### Data and safety monitoring plan

The Data and Safety Monitoring Plan exists to protect the participating subjects [32]. The Principal Investigator (PI) bears responsibility for ensuring participants’ safety on a daily basis. For this study, the National Institute on Aging (NIA) required an Independent Safety Officer, who acts in an advisory capacity to the NIA to monitor participant safety and data collection and evaluate the progress of the study. The NIA Program Officer and the Independent Safety Monitor review the regularly submitted reports to ensure that important information that may affect the safety and welfare of subjects is collected, recognized, and acted upon quickly while still ensuring the validity and integrity of the data. The Independent Safety Officer has no direct involvement with the study, investigation, or intervention.

## Discussion

This single-blinded randomized controlled trial tests whether our adapted CTI program, delivered to community-dwelling older adults who visit the ED for care, decreases revisits within 30 days and, ultimately, costs. It is novel in two different ways. First, the program delivers services through community paramedics, a group rarely used for health promotion or community health activities. Delivery through community paramedics would make this model of care more feasible for widespread adoption, as most communities have paramedics and all communities have ambulance-based care providers with less training (emergency medical technicians). Barriers to program implementation such as lack of nurse or social worker availability would no longer pose problems.

Second, application of the CTI program to the ED-to-home transition, which has a high failure rate, has never been tested. The fact that it is validated in a related setting (hospital-to-home), and that it addresses many of the issues identified as barriers to an effective transition or barriers to accessing care, increases the probability that this intervention will benefit patients [33–39]. More importantly, identifying which patients are helped from this intervention, as determined through our final aim, will provide clear empirical evidence as to which subgroups of patients will reap the benefits of this program.

By collecting demographic, clinical, and outcome data from sources such as patient and caregiver surveys and EHR review, we are positioned to perform extensive data analyses that evaluate the CTI for older adult ED patients. Data analysis will primarily use quantitative methods, as those methods are necessary to achieve our primary aims. However, qualitative content analysis of paramedics’ home visit notes will allow us to explore our findings in greater depth.

There are two main limitations in this study. Although implementation will occur at three sites in two cities rather than a single one, the locations are mid-sized communities with similar characteristics. Therefore, the study’s findings may have only limited external validity with respect to rural or urban settings. Moreover, generalizability may be limited regarding other types of participants (e.g., homeless), participants residing at a larger geographic distance from their treating ED, and other types of CTI coaches (e.g., emergency medical technicians).

In conclusion, the implications and contribution of this project for improving health outcomes for older adults may be great. The study brings the powerful methodology of the randomized controlled trial to bear on the difficult problem of the ED-to-home transition among older adults. It combines the strengths of the validated CTI model with the promise of community paramedicine.

## Abbreviations

CTI: Care transitions intervention
ED: Emergency Department
EHR: Electronic health record
NIA: National Institute on Aging
OR: Odds ratio
PCP: Primary care physician
PI: Principal investigator
